# Circulating Proangiogenic Cells and Proteins in Patients with Glioma and Acute Myocardial Infarction: Differences in Neovascularization between Neoplasia and Tissue Regeneration

**DOI:** 10.1155/2019/3560830

**Published:** 2019-07-21

**Authors:** Karin Huizer, Andrea Sacchetti, Wim A. Dik, Dana A. Mustafa, Johan M. Kros

**Affiliations:** ^1^Department of Pathology, Erasmus Medical Center, Wytemaweg 80, 3015GD Rotterdam, Netherlands; ^2^Department of Immunology, Erasmus Medical Center, Wytemaweg 80, 3015GD Rotterdam, Netherlands

## Abstract

Although extensive angiogenesis takes place in glial tumors, antiangiogenic therapies have remained without the expected success. In the peripheral circulation of glioma patients, increased numbers of endothelial precursor cells (EPCs) are present, potentially offering targets for antiangiogenic therapy. However, for an antiangiogenic therapy to be successful, the therapy should specifically target glioma-related EPC subsets and secreted factors only. Here, we compared the EPC subsets and plasma factors in the peripheral circulation of patients with gliomas to acute myocardial infarctions. We investigated the five most important EPC subsets and 21 angiogenesis-related plasma factors in peripheral blood samples of 29 patients with glioma, 14 patients with myocardial infarction, and 20 healthy people as controls, by FACS and Luminex assay. In GBM patients, all EPC subsets were elevated as compared to healthy subjects. In addition, HPC and KDR^+^ cell fractions were higher than in MI, while CD133^+^ and KDR^+^CD133^+^ cell fractions were lower. There were differences in relative EPC fractions between the groups: KDR^+^ cells were the largest fraction in GBM, while CD133^+^ cells were the largest fraction in MI. An increase in glioma malignancy grade coincided with an increase in the KDR^+^ fraction, while the CD133^+^ cell fraction decreased relatively. Most plasma angiogenic factors were higher in GBM than in MI patients. In both MI and GBM, the ratio of CD133^+^ HPCs correlated significantly with elevated levels of MMP9. In the GBM patients, MMP9 correlated strongly with levels of all HPCs. In conclusion, the data demonstrate that EPC traffic in patients with glioma, representing neoplasia, is different from that in myocardial infarction, representing tissue regeneration. Glioma patients may benefit from therapies aimed at lowering KDR^+^ cells and HPCs.

## 1. Introduction

Although gliomas are among the most vascularized tumors, results of antiangiogenic therapies have been disappointing [1]. Antiangiogenic drugs like Bevacizumab act against VEGF and address sprouting angiogenesis (i.e., the formation of new branches from existing blood vessels). There are various reasons why VEGF blockers like Bevacizumab fail in stopping tumor progression. One reason is that these drugs act against a single step in the complex process of neovascularization that can be compensated for by employing alternative routes of vessel formation [2]. Simultaneously, targeting these alternative routes may result in more successful antiangiogenic therapeutic strategies. Apart from sprouting angiogenesis, circulating endothelial progenitor cells (EPCs) stimulate neovascularization by vasculogenesis, i.e., de novo formation of blood vessels [3–6]. Although these circulating cells are interesting targets for antiangiogenic strategies, there are only scarce data on their frequencies in glioma patients [7]. Since EPCs are involved in physiological tissue repair, therapeutic interventions should ideally not intervene with the normal function of EPCs. EPCs are mobilized by factors secreted by ischemic or neoplastic tissues [8]. Chemoattractants guide EPCs to their target tissues, where they exit from blood vessels and fuel angiogenesis by secreting proangiogenic factors. A subset of EPCs differentiates into endothelial cells and becomes part of the vessel wall [9].

EPCs aid significantly in physiologic tissue regeneration [4]. Following acute myocardial infarction (MI) for instance, EPC subsets are mobilized by the release of proangiogenic factors and chemoattractants [10–13]. HPCs and CD133^+^ cells are engaged in tissue repair following the acute stage of MI [14–16]. In cancer, EPCs participate in tumor vascularization [17, 18], are associated with tumor progression [19], and may diminish the effects of chemotherapy, while blood EPC levels correlate negatively with survival [20]. In the peripheral circulation of both acute MI and (high grade) glioma patients, increased levels of circulating EPCs were demonstrated [7]. While various circulating EPC subsets were studied in the context of MI, limited studies concerning glioma patients are available [7, 21]. Moreover, there are only scarce data correlating the frequency of circulating EPC subsets and the levels of neovascularization-related plasma factors in situations of tissue regeneration and neoplasia [22, 23].

In the present study, we aimed to find new targets for antiangiogenic strategies for glioma patients that would minimally interfere with normal tissue repair. To that aim, we compared the frequency of circulating EPC subsets and plasma levels of a set of chemoattractants, mobilization factors, and angiogenic factors involved in neovascularization in patients with glioma and in patients who suffered from a recent MI. The reason we chose patient with MI to represent the EPC response in acute ischemic tissue repair is the availability of ample literature showing a significant increase in circulating EPCs in this group of patients. We considered including patients with ischemic stroke as a model for EPC response in acute ischemia, but since the literature is much less abundant in this patient group, and since the EPC response in ischemic stroke patients is not unequivocally elevated [7, 24], we decided against this. Blood from healthy adults was used as control. We used an optimized, highly sensitive four-marker-based FACS protocol, allowing for the accurate determination of the EPC subsets [25]. We investigated the frequencies of HPCs (CD34^+^CD133^+/-^CD45^dim⁡^), KDR^+^ cells (KDR^+^CD34^−^CD133^−^), CD133^+^ cells (CD133^+^CD34^−^KDR^−^), KDR^+^CD133^+^ cells (KDR^+^CD133^+^CD34^−^), and circulating endothelial cells (CECs; CD34^bright^KDR^+^CD45^−^CD133^−^).

In addition, we distinguished between CD133^bright^ HPCs, a more primitive phenotype of HPCs that is linked with higher proangiogenic capacity [23, 26, 27], and CD133^−^ HPCs [11].

## 2. Material & Methods

This study was approved by the Medical Ethics Committee of the Erasmus Medical Center, Rotterdam, The Netherlands (MEC-2011-313), and performed in adherence to the Code of Conduct of the Federation of Medical Scientific Societies in the Netherlands (http://www.federa.org/codes-conduct).

### 2.1. Blood Sampling and Handling

Based on a previous study from our group, we anticipated to require between 10 and 25 subjects in each patient and control group to determine statistically significant changes in the frequency of circulating EPC subsets [7]. Since our current study uses more stringent inclusion criteria (treatment-naïve patients with a new diagnosis of glioblastoma, grade II/III astrocytoma, myocardial infarction patients within 1-10 days after acute myocardial infarction) and a much more advanced and fine-tuned FACS protocol [25], we expected that fewer inclusion would suffice to determine statistically significant changes between EPC subsets. For this reason, we aimed to include between 10 and 20 patients in each group of patients and controls.

Blood samples of treatment-naïve patients with radiologically suspected first-episode malignant intracranial tumors were obtained from the Department of Neurosurgery, Erasmus MC. The blood was sampled prior to (diagnostic) surgery and chemo- or radiotherapy. Only patients with a histologically confirmed diagnosis of glioma were included in the current study. In retrospect, out of 38 patients with radiologically suspect malignant intracranial tumors included for FACS analysis, 20 patients received a definitive diagnosis of glioblastoma (GBM), 5 patients of astrocytoma grade II/III (AII/III). Nine patients were diagnosed with brain metastases and 4 patients with various other diagnoses (these 13 patients were excluded from our study). One GBM patient was excluded because of radiotherapy prior to blood sampling and surgery, and one GBM patient was excluded due to technical problems during FACS analysis.

We chose not to group together astrocytoma grade II/III and glioblastoma patients due to the differences between these tumor entities in neovascularization. While in astrocytoma neovascularization is not or only modestly increased and blood vessels are histologically largely similar to normal blood vessels, in glioblastoma there is an extremely high density of blood vessels (up to the point that glioblastomas are among the most vascularized solid tumors), which are haphazardly organized and histologically anomalous (“microvascular proliferation”). We expected that because of this: we could find large differences in the role and frequency of EPCs in the circulation of astrocytoma and glioblastoma patients.

Blood samples from patients who had suffered a recent MI (1-10 days prior to sampling) were received from the Department of Cardiology/Thoracic Center, Erasmus MC. Blood samples from healthy blood donors were obtained via the Sanquin Blood Bank. Age and sex distributions are shown in Tables 1(a) and 1(b). A total of 84 blood samples were included (70 were used for the analysis of chemoattractants and proangiogenic factors and 57 for FACS analysis of EPC subsets). For 43 of the patients, both FACS analysis and plasma marker analysis were carried out. For FACS analysis, we finally included blood samples of 14 MI patients, 18 GBM patients, 5 AII/III patients, and 20 healthy controls (HC). The mean age of GBM patients was 66 years, for MI patients 60 years, and for HC 54 years. GBM patients were significantly older than patients with AII/III (mean ages 66 vs. 45, respectively) reflecting the characteristic age distribution for patients with these tumors.

For each subject, 12-30 ml of venous EDTA blood (BD vacutainer) was collected. Two ml of whole blood was immediately centrifuged at 400 rcf for 10 minutes to isolate platelet-rich plasma (PRP). Next, PRP was centrifuged at 3,000 rcf for 15 minutes. Platelet-poor plasma (PPP) was isolated and stored at -80°C. Blood samples were stored at room temperature in the dark no longer than 18 hours before further FACS analysis.

### 2.2. FACS Analysis

**Figure 1:**
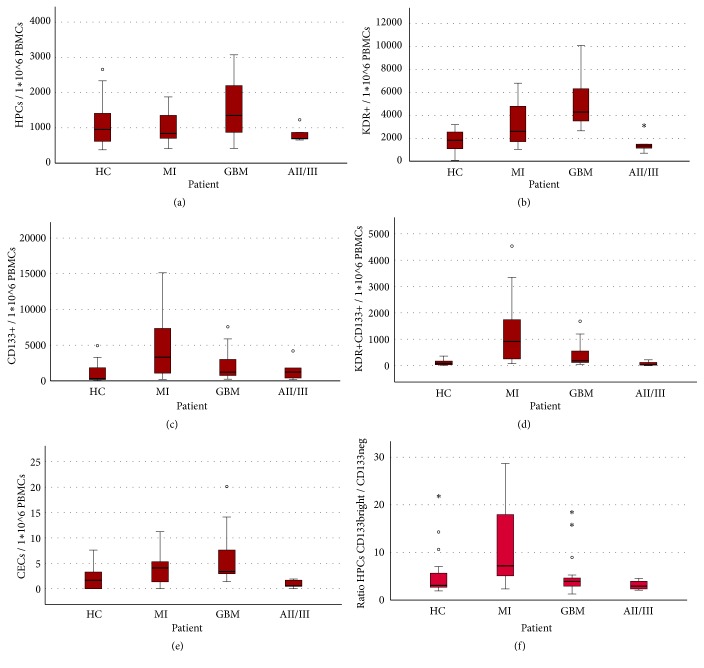
*The frequencies of EPCs in patients included in the study*. Boxplots of frequencies of EPCs (absolute amount in 1*∗*10^6^ PBMCs). Extreme outliers have been excluded from the graphs (extreme outliers excluded: HPCs: 2 (1 MI, 1 GBM); KDR+: 6 (3 MI, 3 HC); CD133+: 2 (1 MI, 1 GBM); KDR+CD133+: 1 GBM). (a)* HPC* levels are the highest in GBM patients. Levels are similar in HC and AII/III patients. (b)* KDR*^*+*^ levels are the highest in GBM and increased in MI patients. Levels are similar in AII/III and HC. (c)* CD13*3^+^ cells are the highest in MI patients and elevated in GBM patients. Levels are similar in AII/III and HC. (d)* KDR*^*+*^*CD13*3^+^ cells are the highest in MI patients and elevated in GBM patients. Levels are similar in AII/III and HC. (e)* CECs* are elevated in both MI and GBM patients. They are indistinguishable between HC and AII/III. (f) The ratio of* CD133*^*bright*^*/CD13*3^−^* HPCs* is highest in MI patients.

HPCs and CECs were analyzed by FACS as described before [25]. Additional gates were set to identify KDR^+^CD34^−^CD133^−^ cells, CD133^+^CD34^−^KDR^−^ cells, and KDR^+^CD133^+^CD34^−^ cells. In brief, peripheral blood mononuclear cells (PBMCs) were isolated from whole blood using Ficoll Paque plus (GE Healthcare). PBMCs were incubated with 10% mouse serum on ice to block aspecific antibody binding. CD34-FITC (Southern Biotech), CD133-PE (MACS Miltenyi), KDR-APC (MACS Miltenyi), and CD45-Viogreen (MACS Miltenyi) were used to stain PBMCs. Cells were washed twice to remove excess antibody and resuspended in FACS sorting buffer (PBS+10% BSA). Hoechst was used as viability dye to exclude dead cells from the analysis. For FACS analysis, we used the BD FACS Aria III. For the initial setup, we analyzed positive control samples using fluorescence minus one as well as isotype controls for every antibody used. We acquired the equivalent of 10-50*∗*10^6^ PBMCs in each analysis using our previously published strategy for the detection of rare cells [25]. We gated the following populations: CD34^+^CD133^+/-^CD45^dim⁡^cells (HPCs), which we subdivided into CD133^negative^, CD133^dim⁡^, and CD133^bright^ subpopulations. In addition, CD34^bright^KDR^+^CD45^−^CD133^−^ cells (CECs) were gated as described in detail in [25]. In addition, CD133^+^ cells (gated as CD34^−^ and KDR^−^), KDR^+^ cells (gated as CD133^−^ and CD34^−^), and KDR^+^CD133^+^ cells (gated as CD34^−^) were analyzed (setup and gating strategy similar to [25]). To quantify subtypes of EPCs, each population was represented as absolute cell numbers in 1*∗*10^6^ CD45^+^ PBMCs. The nonparametric Mann–Whitney U test (SPSS version 24) was used to analyze differences between the groups. Extreme outliers were excluded from the analysis (Figure 1).

### 2.3. Measuring Plasma Chemoattractants and Angiogenic Factors

The concentrations of 21 plasma factors related to EPC biology and neovascularization were measured. The plasma factors were selected based on their key functions in EPC-mediated neovascularization: mobilization and chemotactic factors (CXCL12, CSF2, and CSF3), de-adhesion and invasion factors (MMP2, MMP9), and proangiogenic factors/microenvironment regulators (VEGFA, KITL, vWF, EGF, FGFb, EPO, Ang2, Ang1, BDNF, VCAM1, PDGFBB, tenascin-c, periostin, HGF, and PGF) [22, 28–33]. The angiogenic factors either directly stimulate angiogenesis or represent regulators of angiogenesis like MMP-2, MMP-9, tenascin-c, and periostin that aid in generating a microenvironment favoring neovascularization. The functional delineations are, however, not strict and there is extensive overlap in functions of the factors. The plasma factors were measured in PPP using 3 different custom-mixed magnetic bead-based MAGPIX®-Luminex assays from R&D (see Additional File 1). Analyses were performed on PPP, diluted as recommended by the company (R&D Systems, Abingdon, UK). Because of low concentrations, the levels of CSF2, CSF3, vWF, VEGF, EGF, and CXCL12 were measured by their raw mean fluorescence intensity (MFI) values. In order to determine whether using MFI values yielded reliable statistical results, we compared calculated concentrations of markers with a high concentration, with their corresponding MFI values. This yielded identical statistical results. In addition, the results of the low concentration markers (using their MFI values) fit with preexisting literature [34]. Therefore, the MFI values of these markers were added to the data set. The nonparametric Mann–Whitney U test (SPSS version 24) was used to analyze differences between the groups.

### 2.4. Correlating Plasma Factors with EPC Frequencies in GBM and MI

To determine if the levels of chemoattractants and mobilization factors were related to EPC and CEC levels, we conducted correlation analyses. Since the frequencies of EPCs display a non-Gaussian distribution and since the correlation between EPC frequencies and plasma factors proved to be nonlinear, we used Spearman's rho to calculate correlation coefficients.

## 3. Results

### 3.1. EPC Absolute Frequencies

**Figure 2:**
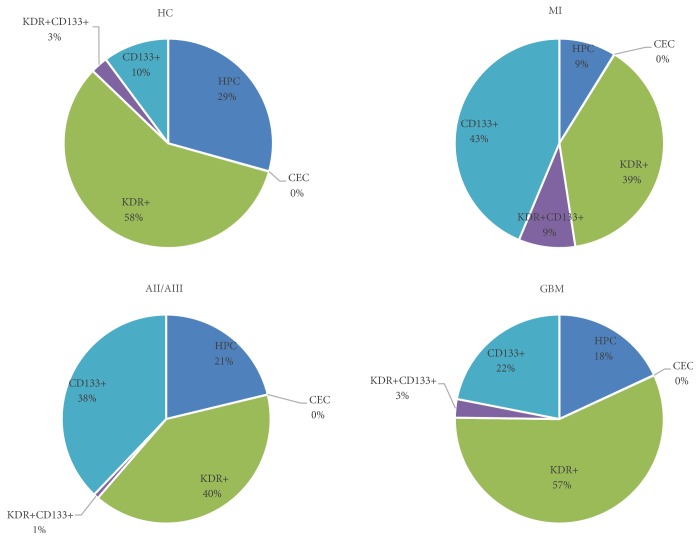
*Relative percentages of EPCs*. Relative percentages of EPCs (median values) by patient group are shown in pie charts.

**Figure 3:**
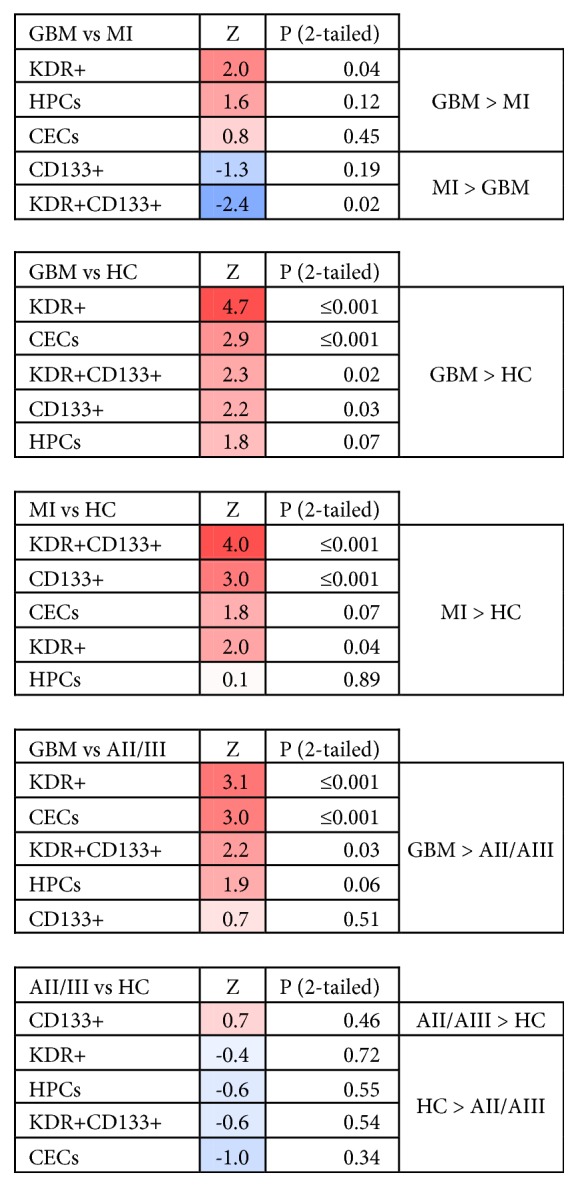
*Differences in EPC frequencies between patients*. EPC levels were represented as absolute cell numbers in 1*∗*10^6^ CD45^+^ PBMCs. The nonparametric Mann–Whitney U test (SPSS version 24) was used to analyze differences between the groups (p-values are 2-tailed). Direction of Z-score was adjusted as follows: negative to positive when GBM levels were higher than HC/MI/AII/AIII and when MI levels were higher than HC. The heat-maps are based on the levels and directions of Z-scores (red indicated higher levels of EPCs; blue indicated lower levels of EPCs in each comparison). KDR^+^: KDR^+^CD34^−^CD133^−^ cells. CD133^+^: CD133^+^CD34^−^KDR^−^ cells. KDR^+^CD133^+^: KDR^+^CD133^+^CD34^−^ cells. HPCs: CD34^+^CD133^+/-^CD45^dim⁡^. CECs: CD34^bright^KDR^+^CD45^−^.

In all groups HPCs, KDR^+^ and CD133^+^ cells represented the majority of circulating EPCs (Figure 2). In patients with GBM and acute MI, all EPC subsets were higher as compared to HC, except for the HPC fraction in MI (Figures 1 and 3). In GBM patients, KDR^+^ (Z=-2.0; p=0.04) and HPC levels (Z=-1.6; p=0.12) were higher as compared to those in MI patients, while in MI patients CD133^+^ (Z=-1.3; p=0.19) and KDR^+^CD133^+^ (Z=-2.0; p=0.02) levels exceeded those in GBM patients.

### 3.2. EPC Relative Fractions

The relative fractions of the EPCs differed in the groups (Figure 2). In GBM, the largest fraction of EPCs was KDR^+^ (57%), while in MI patients the largest fraction was CD133^+^ cells (43%). In addition, in GBM, the HPC fraction was twice as big as in MI, while in MI, the KDR^+^CD133^+^ fraction was three times larger than in GBM patients. The relative fractions of EPCs in HC were similar to those in GBM. However, the absolute numbers of circulating EPCs are significantly elevated in GBM patients (Figure 1). Noticeably, absolute levels of EPCs in AII/III patients were comparable to HC, while the relative distribution of EPC subsets was very different: in AII/III the fraction of CD133^+^ cells was significantly larger and that of HPCs was significantly smaller than in HC. Comparing AII/III with GBM, we found the KDR^+^ fraction increased along with malignancy grade from 40% in AII/III to 57% in GBM. The CD133^+^ cell fraction decreased from 38% in AII/III to 22% in GBM.

The KDR^+^CD133^+^ fraction in all groups was relatively small (for all groups below 10%) and CECs were the smallest population, with percentages below 1% for all groups.

### 3.3. Plasma Factors

**Figure 4:**
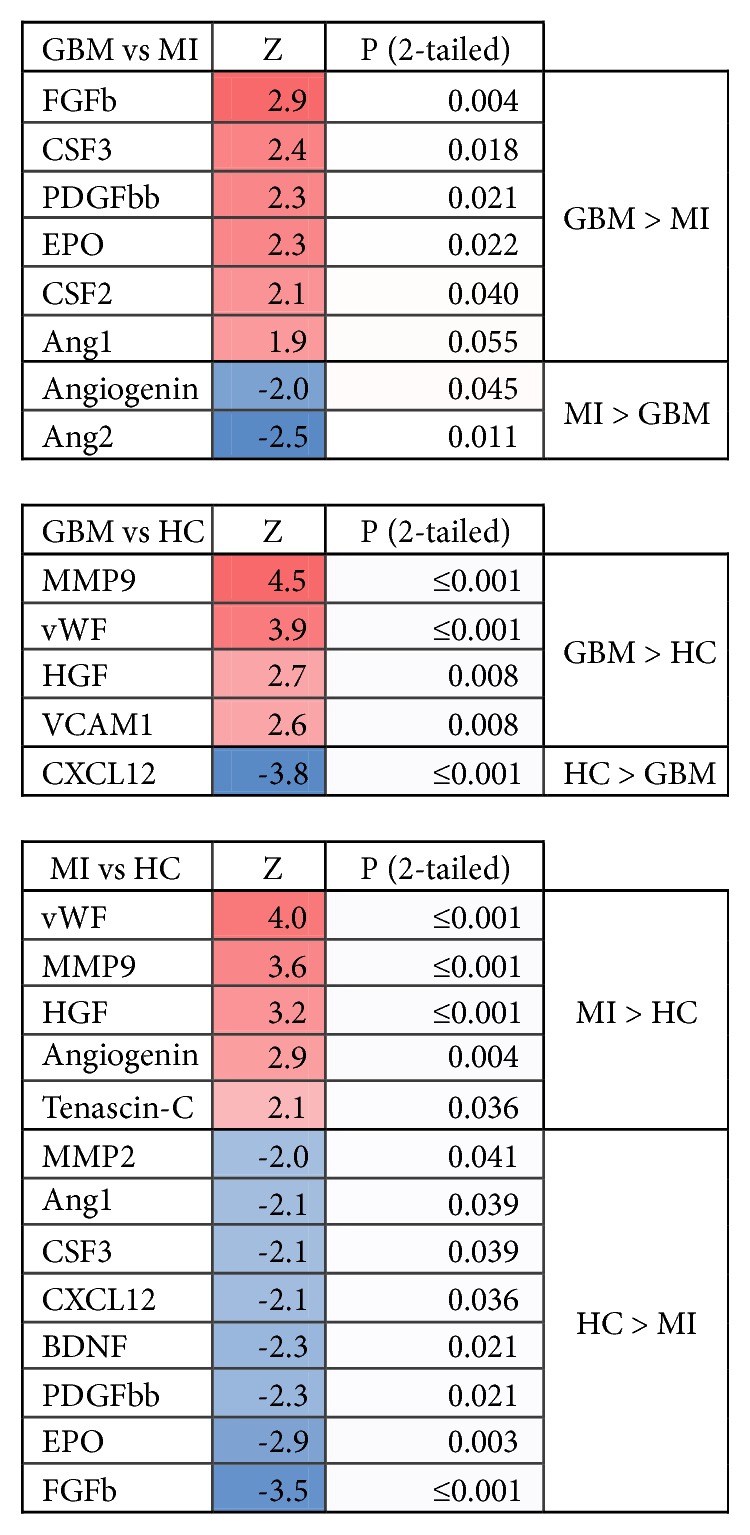
*Differences in levels of plasma factors between patients*. Z-scores and p-values of differences in the levels of plasma factors between patient and control groups (nonparametric Mann–Whitney U test). Direction of Z-score was adjusted as follows: negative to positive when GBM plasma levels of the factors were higher than MI/HC and when MI levels were higher than HC. The heat-maps are based on the levels and directions of Z-scores (red indicated higher levels of plasma factors; blue indicated lower levels of plasma factors in each comparison).

There were considerable differences in the concentrations of the various plasma factors between the groups (Figure 4). Both in GBM and MI patients, the factors MMP9, HGF, and vWF were elevated in plasma relative to HC. VCAM1 was specifically elevated in GBM, while angiogenin and tenascin-c were specifically elevated in MI, relative to HC. Nine factors were higher in HC than in MI patients and only one factor, CXCL12, was higher in HC than in GBM patients. Most plasma angiogenic factors were higher in GBM than in MI patients. Ang2 and angiogenin levels were higher in MI patients compared to GBM, while CSF2, CSF3, FGFb, EPO, PDGFBB, Ang1, and the ratio Ang1/Ang2 were all higher in GBM than in MI patients. Interestingly, the concentrations of plasma factors in AII/III patients were indistinguishable from HC, except for CXCL12, which was decreased in AII/III. See Figure 4.

### 3.4. Correlations Between Plasma Factors and EPC Frequencies in GBM and MI

**Figure 5:**
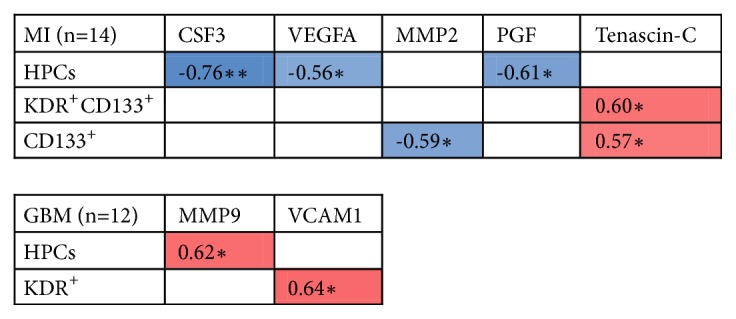
*Correlation between plasma factors and EPC subtypes*. *∗∗*Correlation is significant at the 0.01 level (2-tailed). *∗*Correlation is significant at the 0.05 level (2-tailed). We used Spearman's rho to calculate correlation coefficients between plasma factor and EPC subtype levels. Figure 5 shows Spearman's rho for significant correlations between EPC levels and plasma factor levels in MI and GBM patients. Blue color indicates a negative correlation between plasma factor and EPC frequency; red indicates a positive correlation. For a complete overview (including CD133^bright^ and CD133^−^ HPC subtypes and correlations between EPC frequencies and plasma factors in all samples grouped together), see Additional File 2.

The Spearman correlations between EPC subpopulations and plasma factors in patients with gliomas, MI, and HC are shown in Additional File 2. In GBM patients, MMP9 correlated strongly with HPC levels (rho=0.62; p=0.03) and KDR^+^ levels correlated with VCAM1 plasma concentration (rho=0.64; p=0.04). In MI patients, HPC levels correlated negatively with plasma concentrations of CSF3 (rho=-0.76; p=0.002), VEGFA (rho=-0.56; p=0.04), and PGF (rho=-0.61; p=0.02). CD133^+^ levels correlated negatively with MMP2 plasma concentration (rho=-0.59; p=0.03), while tenascin-c concentration correlated positively with both KDR^+^CD133^+^ levels (rho=0.60; p=0.03) and CD133^+^ levels (rho=0.57; p=0.03). Significant correlations for GBM and MI are shown in Figure 5.

## 4. Discussion

We compared circulating EPC populations and plasma factors of patients with GBM and MI to pinpoint potential differences in EPC biology that may lead to the development of new therapeutic strategies directed against glioma-specific neovascularization.

While there was a general elevation of EPC levels in both GBM and MI patients compared to HC, we found differences in specific EPC subsets between GBM and MI patients. In GBM patients, HPCs and KDR^+^ cells were elevated compared to MI patients. In MI patients, KDR^+^CD133^+^and CD133^+^ cells were higher than in GBM patients. Increased levels of CD133+ cells were described before in MI patients [35]. An increase in KDR^+^CD133^+^ cells was reported following vascular damage due to burns or surgery [36], as well as in GBM and patients with other tumors [19, 37]. Data on circulating KDR^+^(CD34^−^CD133^−^) cells are largely lacking in the literature. Increased levels of circulating KDR^+^ bone-marrow-derived EPCs were reported in a cancer mouse model [38], which is compatible with our findings in glioma patients. Increased HPC levels were observed previously in untreated GBM patients [39], while levels seem to normalize and even decrease following treatment [40]. In order to refine the HPC populations, three subgroups of these cells are distinguished: CD133^−^, CD133^dim⁡^, and CD133^bright^ [25]. In the present study, we found a significant increase in the ratio of CD133^bright^/CD133^−^ HPCs in patients with MI, compared to GBM patients (Figure 1(f)). The more primitive phenotype of CD133^bright^ HPCs is reportedly linked with higher proangiogenic capacity of these cells as compared to CD133^−^ cells [23, 26, 27]. An increase in CD133^+^ HPCs is seen in acute MI [41], while levels of these cells are low in patients with chronic vascular disease (low CD133^bright^/CD133^−^ HPC ratio [26]), suggesting that the rise in CD133^bright^/CD133^−^ HPC ratio is linked to acute ischemia.

There are various explanations for the numerical differences in EPC subsets between patients with GBM and MI. Both conditions are associated with increased neovascularization. One explanation is that MI represents a situation of acute injury, followed by programmed regeneration, while in neoplasia such as GBM, acute ischemic events due to, e.g., vessel thrombosis, occur on top of a background of chronic hypoxia and neoplastic vascular remodeling. In acute MI, a time course for EPC and CEC dynamics exists: within hours after MI, a peak in CECs appears in the bloodstream, which declines over the following weeks [36, 42]. Over the course of 3-7 days, CD133^+^ cells increase, peaking around day 7, a phenomenon that was consistent with the present analysis [35]. Subsequently, somewhat later than CD133^+^ cells, HPC levels rise [10, 13, 35]. The increase in the levels of both CD133^+^ cells and CD133^+^KDR^+^ cells in MI patients suggests that these cells are influenced by similar regulatory mechanisms and that these EPC subtypes are particularly important in the early phase of acute ischemia. Elevated levels of CD133^+^ cells have been described before in MI and GBM and encompass large part of the HPC population, since in these studies no further separation of EPC subtypes was made [35, 43, 44].We found that the absolute levels of EPCs and CECs were increased in MI and GBM, but not in the astrocytomas grade II and III, reflecting the low level of neovascularization in lower-grade gliomas.

The finding of higher levels of CECs in patients with GBM and MI compared to patients with lower-grade gliomas is corroborated by literature on patients with MI and neoplasia, including gliomas [45–53]. The lower levels of CECs in patients with gliomas of lower malignancy grades, in which neovascularization is less abundant, supports the notion that CEC levels correspond with the degree of vessel formation and remodeling in cancer. So far, the presence of CECs was considered to passively reflect vessel wall damage only, but there are indications that a viable subset should be considered as cells with potent proangiogenic and vasculogenic capacities [25, 54]. These cells give rise to outgrowth endothelial cells (OECs) when brought in cell culture and strongly stimulate neovascularization, incorporate in the vessel wall, and home to malignant tumors [55–57]. Increased levels of OEC precursor cells correlate with a better prognosis for patients with MI and coronary artery bypass grafts, illustrative of their proangiogenic capacities [36, 58]. Conversely, higher (viable) CEC levels correspond with a worse prognosis for patients with GBM [50, 51, 59] and other cancers [53, 60, 61]. Therefore, CECs may be considered as potential therapeutic targets in both cancer and infarction.

Limitations to any study on circulating EPCs in human subjects include difficulties of comparing study results to the literature, due to the lack of a clear and comparable definition of EPC subsets and the use of different techniques to determine or isolate EPCs. This makes it challenging to compare findings of different studies into EPCs. For instance, Stamm et al. [62] used magnetic beads to isolate CD133^+^ cells from bone marrow aspirates of myocardial infarction patients undergoing subsequent coronary artery bypass graft. The CD133^+^ bone marrow cells would in our study translate into a mixture of CD133^+^ HPCs, CD133^+^KDR^−^CD34^−^ cells and CD133^+^KDR^+^CD34^−^ cells. Which of these different subsets will have been accountable for the beneficial effect in the study of Stamm et al. remains to be determined.

The KDR^+^CD34^−^CD133^−^ population in the present study was not described before in the literature. However, this population needs to be distinguished from CECs (CD34^++^KDR^+^CD45^−^) and from CD133^+^KDR^+^ EPCs. Other studies have found increased levels of CECs and CD133^+^KDR^+^ cells in MI patients [36, 45, 63]. Interestingly, we found low levels of CD34 expression in some KDR^+^CD34^−^CD133^−^ sorted populations (data not shown), suggesting that the expression of CD34 may have been too low to detect by FACS and suggesting a relationship with the more frequently described KDR^+^CD34^+^ EPC population in the literature. In our study, the KDR^+^CD34^−^CD133^−^population was exclusively CD45^+^ indicative of hematopoietic lineage. We also found high expression of proangiogenic factors in these cells (data not shown). Therefore, we believe that the KDR^+^CD34^−^CD133^−^ EPC subset stimulates neovascularization just like other EPC subsets. Other confounders to human EPC-related studies are differences in age of subjects included. Younger age is associated with higher levels of circulating EPCs [64]. We do not believe, however, that the slight difference in age has influenced the results in GBM vs. MI patients (Table 1). AII/III patients are younger than GBM and MI patients, reflecting the age difference in the occurrence of these tumors. Young age is associated with higher circulating levels of EPCs. The significantly lower levels of EPCs in AII/III patients vs. GBM and MI patients emphasize the strong effects of underlying pathology on the EPC levels. In addition, sex differences may associate with circulating EPCs levels that vary based on menstrual phase in premenopausal women [65]). Unlike the situation in the glioma group, in the MI group, males predominated. However, since most, if not all, women in this study will have been postmenopausal (based on age), we do not believe that sex will have had a significant influence on the results either. Other confounders like physical exercise status were not controlled for. High-intensity physical exercise may lead to peaks in circulating EPC and CEC levels. This could be an explanation for high EPC level outliers in our study, particularly in the healthy control group. Other explanations for outliers can be time after MI (we included MI patients 1-10 days after myocardial infarction; within this timeframe, the dynamics of EPC and CEC levels can vary), GBM tumor characteristics (size, level of neovascularization), and medication use (e.g., statins can increase the levels of circulating EPCs or normalize previously reduced levels of EPCs in the context of chronic vascular disease and improve their function [66]).

The presence of the blood-brain barrier (BBB) or blood-tumor barrier in the case of GBM is highly unlikely to form an anatomical barrier relevant for EPCs. EPCs do not need to cross the BBB into the brain parenchyma to exert their angiogenic and vasculogenic effects. EPC entrance into the Virchow-Robin space, directly surrounding blood vessels, would suffice for the promotion of angiogenesis through the production of proangiogenic factors. No entrance of EPCs into the brain parenchyma is required for this process. Further, the BBB is severely impaired in glioblastoma, allowing cells to freely enter the brain [67]. Besides, even an intact BBB would allow for the selective entrance of (inflammatory) cells from the periphery into the parenchyma [68].

Since factors secreted by the target tissues are essential for the recruitment and function of EPCs, we investigated a panel of mobilization factors, chemoattractants, and angiogenic factors in plasma along with EPC levels and found significant differences in their mean concentrations between the patient groups and controls. Elevated levels of these factors were previously reported in blood and tumor tissue of patients with GBM [69–75] and of patients with MI [76–83]. Because the levels of vWF, MMP9, VCAM1, angiogenin, and HGF were increased in both GBM and MI patients, but not in the lower-grade gliomas, these factors seem to be necessary for neovascularization in general, both under reactive and high-grade neoplastic conditions. Together with VEGFA, these factors were higher in GBM as compared to the lower-grade gliomas, illustrative of their correlation with tumor grade and level of glioma neovascularization. Increased concentrations of vWF in GBM patients were previously reported [42]. Interestingly, in MI patients, many of the factors were decreased as compared to HC (Figure 4). This may in part be a reflection of chronic cardiovascular disease and vascular dysfunction preceding the acute infarction, as some circulating factors are already reduced in (un)stable angina [84, 85]. An increase in levels when acute ischemia ensues could then still remain below normal levels [86]. The increased levels of tenascin-c, vWF, MMP9, VCAM1, and angiogenin may reflect the response to acute ischemia. Angiogenin increases after MI, but is not elevated in patients suffering from stable cardiovascular disease [82]. Only angiogenin and angiopoietin-2 were increased in MI patients compared to GBM patients, suggestive of their association with the acute onset of ischemia occurring in MI. CXCL12 is one of the main mobilization factors for HPCs and other EPCs. Surprisingly, CXCL12 levels were lower in all patient groups relative to healthy controls. Reduced CXCL12 levels were reported in patients with MI previously [87–89] and also in experimentally induced MI in mice [88]. Our finding of low CXCL12 levels in GBM patients seems to conflict with literature data, where CXCL12 levels allegedly correlate positively with glial tumor progression [37, 50, 90]. The discrepancies may be explained by concurrent treatment, for instance, with antiangiogenic agents [50] in these studies, whilst in our study GBM patients were treatment-naïve.

We correlated the concentrations of mobilization factors and chemoattractants with the levels of EPC subsets in order to investigate a potential relationship between circulating levels of cells and factors. We found various correlations between the plasma factors on the one hand and the EPC subsets on the other hand (Figure 5). Interestingly, in MI patients, tenascin-c levels correlated positively with CD133^+^ and KDR^+^CD133^+^ levels. Tenascin-c is a matricellular protein which is upregulated in ischemic myocardial tissue and aids in recruiting EPCs to the infarcted area [91]. Notably, plasma levels of tenascin-c are increased in the acute phase of MI [92, 93] corresponding to the early phase in which CD133^+^ cells are released. A potential effect of plasma tenascin-c on the mobilization of EPCs, however, remains speculative.

In GBM patients, plasma levels of MMP9 correlated positively with HPC frequencies, which seems in line with data suggesting that MMP9 can mobilize HPCs from the bone marrow [94]. Increased levels of CECs and vWF and VCAM-1 are known to represent vessel damage and activated endothelial cells, thus explaining their elevation in GBM patients.

How could our findings eventually be translated to novel therapeutic targets for GBM patients? From a therapeutic perspective, several different approaches could be chosen: firstly, by targeting the mobilization factors that lead to higher KDR^+^ (and other EPC) levels in GBM patients. We found a strong positive correlation between plasma VCAM1 levels and KDR^+^ EPCs in GBM. Should further studies indicate that VCAM1 can act as a mobilization factor for KDR^+^ EPCs, anti-VCAM1 antibodies could potentially reduce circulating KDR^+^ EPC levels in GBM patients. We found a strong positive correlation between plasma MMP9 levels and circulating HPC levels in GBM patients. From the literature, a causal relationship between the two can be assumed since MMP9 is a known mobilization factor for HPCs (and possibly other EPCs) [94]. Strategies to reduce plasma MMP9 levels could decrease circulating HPC (and possibly other EPC) levels in GBM patients. Likewise, with more of these causal relations between plasma factors and EPC levels coming to light, more therapeutic strategies of a similar nature can be generated.

Contrarily, in MI patients, the same strategies could be used in an opposite fashion: administering mobilization factors with the aim of increasing levels of circulating EPCs (e.g., we found a strong positive correlation between plasma tenascin-C levels and circulating levels of KDR^+^CD133^+^ and CD133^+^ EPCs; should tenascin-C prove to act as a mobilization and/or homing factor to these EPCs, increasing the level of circulating and/or myocardial tissue tenascin-C could be beneficial to EPC mobilization and homing to hypoxic myocardial tissue).

Secondly, the homing mechanisms of EPCs to their target tissue can be therapeutically manipulated. In the case of GBM, homing factors such as CXCL12 could be increased in plasma (leading to a reduced gradient of GBM tissue-to-blood CXCL12 levels and potentially reduced homing of EPCs to target GBM tissue; this hypothesis would, obviously, need to be carefully tested in further studies). Another option could be to implant a device that captures KDR^+^ (and other) EPCs from the circulation of GBM patients, thereby preventing them from reaching GBM tissue and exerting their proangiogenic effect (a similar strategy is used in preclinical studies in MI patients with EPC-capturing stents to increase neovascularization [95]). To the best our knowledge, this strategy has not been tested with the aim of decreasing circulating levels of EPCs (and decreasing their homing efficiency to tumor tissue) in cancer patients yet, but could be promising.

Thirdly, the ability of EPCs to migrate to GBM tumor tissue means that EPCs themselves could be used as vessels for transport of cancer-blocking agents to the tumor (e.g., radioactive or chemotherapeutic compounds). Whether there is a difference between EPC subsets in their ability to migrate to GBM tumor tissue remains to be determined (e.g., are KDR^+^ EPCs better able to home to GBM tissue than other EPCs? If so, this cell type could preferentially be used for this strategy). This hypothesis has been postulated before in the literature [96]. Contrarily, in the case of MI, (KDR^+^CD133^+^, CD133^+^KDR^−^) EPCs could be altered (in vitro) to, e.g., express higher levels of proangiogenic factors and readministered to MI patients to aid in tissue recovery.

## 5. Conclusion

In conclusion, while neovascularization in both the context of high-grade neoplasia (GBM) and acute ischemia (MI) is associated with a rise in EPC levels, we found differences in their relative EPC subsets. Our findings indicate that the process of EPC-related neovascularization differs between these two diseases. The data are supportive of the development of EPC targeted therapeutic strategies that differ in both contexts. In acute ischemic conditions, stimulation of EPC-induced neovascularization is needed (increasing the circulating levels of KDR^+^CD133^+^ and CD133^+^ cells). However, in GBM, inhibition of EPC-induced neovascularization is necessary (specifically focusing on decreasing KDR^+^ cells and HPCs).

## Figures and Tables

**(a):** 

*FACS*		*Age*				*Sex*
*Samples*	*N*	*Mean*	*SD*	*Minimum*	*Maximum*	*m/f*
*HC*	20	54	12	22	69	15/5

*MI*	14	60	11	38	77	11/3

*AII/III*	5	45	11	32	58	0/5

*GBM*	18	66	10	45	79	9/9

*Total*	57					

**(b):** 

*Luminex*		*Age*				*Sex*
*Samples*	*N*	*Mean*	*SD*	*Minimum*	*Maximum*	*m/f*
*HC*	20	59	7	38	69	15/5

*MI*	14	60	11	38	77	11/3

*AII/III*	7	53	12	35	69	2/5

*GBM*	29	65	9	45	81	16/13

*Total*	70					

## Data Availability

The datasets used and/or analyzed during the current study are available from the corresponding author upon reasonable request.

## Abbreviations

AII/AIII: Astrocytoma grade 2 and grade 3
Ang1: Angiopoietin 1
Ang2: Angiopoietin 2
BDNF: Brain-derived neurotrophic factor
CEC: Circulating endothelial cell
CSF2: Colony stimulating factor 2
CSF3: Colony stimulating factor 3
CXCL12: CXC-motif chemokine ligand 12
EDTA: Ethylenediaminetetraacetic acid
EGF: Epidermal growth factor
EPC: Endothelial progenitor cell
EPO: Erythropoietin
FACS: Fluorescence-activated cell sorting
FGFb: Basic fibroblast growth factor
GBM: Glioblastoma
HC: Healthy control
HGF: Hepatocyte growth factor
HPC: Hematopoietic progenitor cell
KDR: Kinaseinsert domain
KITL: KIT-ligand
MFI: Mean fluorescence intensity
MI: Myocardial infarction
MMP2: Matrix metalloproteinase 2
MMP9: Matrix metalloproteinase 9
PBMC: Peripheral blood mononuclear cell
PDGF-BB: Platelet-derived growth factor BB
PGF: Placental growth factor
PPP: Platelet-poor plasma
PRP: Platelet-rich plasma
VCAM1: Vascular cell adhesion molecule 1
VEGFA: Vascular endothelial growth factor A
vWF: von Willebrand factor.

## Supplementary Materials

Supplementary Materials
* Additional File 1: Luminex Assays. *Luminex Assays (customized) were used in the study. Three separate assays were included. Columns show the factors analyzed, the dilution factor, bead region used, and value (pg/ml) of standard. Type of factor (AF: angiogenic factor, Mob: mobilization factor, Chemo: chemoattractant) is specified.* Additional File 2: Correlation between Plasma Factors and EPC Subtypes. *We used Spearman's rho to calculate correlation coefficients between plasma factor and EPC subtype levels. All samples were analyzed, as well as every group separately. Green highlighted correlations indicate p-values<0.05.
