# Supplementary material for: Circulating Proangiogenic Cells and Proteins in Patients with Glioma and Acute Myocardial Infarction: Differences in Neovascularization between Neoplasia and Tissue Regeneration
Source: J Oncol. 2019 Jul 21;2019:3560830. doi: 10.1155/2019/3560830 (PMC6679840; doi:10.1155/2019/3560830)
Supplement: Supplementary Materials — Additional File 1: Luminex Assays. Luminex Assays (customized) were used in the study. Three separate assays were included. Columns show the factors analyzed, the dilution factor, bead region used, and value (pg/ml) of standard. Type of factor (AF: angiogenic factor, Mob: mobilization factor, Chemo: chemoattractant) is specified. Additional File 2: Correlation between Plasma Factors and EPC Subtypes. We used Spearman's rho to calculate correlation coefficients between plasma factor and EPC subtype levels. All samples were analyzed, as well as every group separately. Green highlighted correlations indicate p-values<0.05. [file 3560830.f1.docx]

**Additional File 1: Luminex Assays**

Legends: Luminex Assays (customized) used in the study. Three separate assays were included. Columns show the factors analyzed, the dilution factor, bead region used, and value (pg/ml) of standard. Type of factor (AF: angiogenic factor, Mob: mobilization factor, Chemo: chemoattractant) is specified.

|  | **Markers** | **dilution factor PPP** | **Region** | **Value (pg/ml) Standard** | **Involved in (function)** |
| --- | --- | --- | --- | --- | --- |
| **Assay I** | vWF | 1:2 | 15 | 23,6 | AF |
|  | VCAM-1 |  | 57 | 2336,000 | AF |
|  | VEGFA |  | 26 | 1,970 | AF/Chemo |
| **Assay II** | BDNF | 1:2 | 15 | 5,300 | AF |
|  | SDF1a |  | 20 | 3,200 | MOB/AF/Chemo |
|  | ANG-2 |  | 26 | 29,175 | AF |
|  | EPO |  | 34 | 111,000 | AF |
|  | GM-CSF |  | 46 | 12,800 | MOB/Chemo |
|  | G-CSF |  | 54 | 8,150 | MOB/Chemo |
|  | ANG-1 |  | 64 | 28,400 | AF |
|  | PLGF |  | 72 | 2,800 | AF |
|  | PDGF-BB |  | 18 | 2,600 | AF |
|  | EGF |  | 25 | 4,440 | AF |
|  | SCF |  | 27 | 14,500 | AF/Chemo |
|  | Tenascin-C |  | 35 | 10,200 | AF |
|  | FGF-basic |  | 47 | 6,540 | AF |
|  | Periostin |  | 55 | 293,900 | AF |
|  | HGF |  | 66 | 9,320 | AF |
| **Assay III** | MMP-9 | 1:50 | 14 | 37,370 | MOB/AF |
|  | Angiogenin |  | 66 | 6,250 | AF |
|  | MMP-2 |  | 20 | 54,080 | MOB/AF |
